# Building a protein interaction (PIN) database for autism candidate genes

**DOI:** 10.1186/gb-2011-12-s1-p22

**Published:** 2011-09-19

**Authors:** Catherine Croft Swanwick, Sharmila Banerjee-Basu

**Affiliations:** 1 MindSpec, McLean, VA 22181, USA

## Background

Hundreds of diverse genetic loci have been linked to autism spectrum disorders (ASDs), making large-scale analysis essential for understanding the molecular events underlying the pathogenesis of these disorders. Our laboratory first released the autism database AutDB in 2007 as a bioinformatics tool for systematic curation of all known ASD candidate genes [1-3]. AutDB was designed with a systems biology approach, integrating genetic entries within the Human Gene module with corresponding behavioral, anatomical and physiological data in the Animal Model module. In June 2011, we released a new Protein Interaction (PIN) module of AutDB, which serves as a comprehensive, up-to-date resource on the direct protein interactions of ASD-linked genes.

## Methods

To curate the PIN module, our researchers utilize a multi-level annotation model to systematically search, collect and extract information entirely from published, peer-reviewed scientific literature. Although we initially consult public molecular interaction databases (HPRD and BioGRID) and commercial molecular interaction software (Pathway Studio, version 7.1), every interaction is manually extracted and verified by evaluating the primary reference articles from PubMed. Our manual curation has proved critical for accurate annotation, because these references were the second largest source of references for the initial PIN dataset, providing more interactions than both HPRD and Pathway Studio. Each ASD gene entry within the PIN module is presented as a multi-level display, with interactive graphical and tabular views of its corresponding interactome.

## Results

The initial PIN dataset includes interactomes for 86 ASD candidate genes, with a total of 1,311 direct protein interactions garnered from 533 unique primary references. These interactomes are composed of 6 interaction types and 13 species, documented by 402 distinct pieces of evidence. Our researchers will expand and maintain the data content of the PIN module with systematic updates.

## Conclusions

We have created an integrated bioinformatics tool that can be used for the large-scale analysis of the biological relationships among ASD candidate genes. Such network analysis is envisioned to provide a framework for identifying the key molecular pathways underlying ASD pathogenesis, potentially leading to the development of novel drug therapies.
